# WDR76 Co-Localizes with Heterochromatin Related Proteins and Rapidly Responds to DNA Damage

**DOI:** 10.1371/journal.pone.0155492

**Published:** 2016-06-01

**Authors:** Joshua M. Gilmore, Mihaela E. Sardiu, Brad D. Groppe, Janet L. Thornton, Xingyu Liu, Gerald Dayebgadoh, Charles A. Banks, Brian D. Slaughter, Jay R. Unruh, Jerry L. Workman, Laurence Florens, Michael P. Washburn

**Affiliations:** 1 Stowers Institute for Medical Research, Kansas City, MO, 64110, United States of America; 2 Department of Pathology and Laboratory Medicine, The University of Kansas Medical Center, 3901 Rainbow Boulevard, Kansas City, Kansas, 66160, United States of America; The University of Hong Kong, HONG KONG

## Abstract

Proteins that respond to DNA damage play critical roles in normal and diseased states in human biology. Studies have suggested that the *S*. *cerevisiae* protein CMR1/YDL156w is associated with histones and is possibly associated with DNA repair and replication processes. Through a quantitative proteomic analysis of affinity purifications here we show that the human homologue of this protein, WDR76, shares multiple protein associations with the histones H2A, H2B, and H4. Furthermore, our quantitative proteomic analysis of WDR76 associated proteins demonstrated links to proteins in the DNA damage response like PARP1 and XRCC5 and heterochromatin related proteins like CBX1, CBX3, and CBX5. Co-immunoprecipitation studies validated these interactions. Next, quantitative imaging studies demonstrated that WDR76 was recruited to laser induced DNA damage immediately after induction, and we compared the recruitment of WDR76 to laser induced DNA damage to known DNA damage proteins like PARP1, XRCC5, and RPA1. In addition, WDR76 co-localizes to puncta with the heterochromatin proteins CBX1 and CBX5, which are also recruited to DNA damage but much less intensely than WDR76. This work demonstrates the chromatin and DNA damage protein associations of WDR76 and demonstrates the rapid response of WDR76 to laser induced DNA damage.

## Introduction

In the human body, tens of thousands of DNA lesions occur each day [1]. These lesions, if not repaired or repaired incorrectly, produce mutations or wider-scale genome aberrations that can lead to many different diseases. Many of the side effects of cancer therapy, such as hair loss, nausea, and bone marrow suppression are also caused by DNA damage [2]. Therefore, identifying factors associated with a particular type of DNA damage response is of great importance towards the understanding of these biological processes and diseases. We previously described a poorly characterized protein in *S*. *cerevisiae* named CMR1/YDL156w that associated strongly both with histones and with DNA repair and replication proteins [3]. Additional studies on yeast CMR1/YDL156w have demonstrated that the protein had higher affinity for UV-damaged DNA and had increased localization to chromatin upon UV treatment [4]. Recently, Jones *et al*. have shown that YDL156w is recruited to coding regions of transcribed regions in yeast and may regulate RNA Polymerase II occupancy [5]. In a screen for proteins involved in the DNA damage response, Tkach *et al* found CMR1/YDL156w localized to foci distinct from the known Rad52 foci [6]. In addition, a computational analysis of co-expressed genes from cell cycle regulated yeast genes found CMR1/YDL156w clustered with DNA-metabolic process genes [7]. Recently, an in depth investigation of CMR1/YDL156w revealed links to chromatin, localization into foci, a response to replication stress, and involvement in a DNA damage checkpoint [8].

Less is known about the human homologue of CMR1/YDL156w, WDR76. Intriguingly WDR76 was found to associate with the Cul4-DDB1 ubiquitin ligase complex [9] and was also found in a screen for 5-(hydroxyl)methylcytosine readers [10]. Spruijit *et al*. purified GFP-WDR76 and found interactions with SPIN1 (also known as OCR), HELLS, and GAN [10], and Gallina *et al*. reported GFP-WDR76 to be associated with HELLS, XRCC5, and XRCC6 [8]. In addition, Gallina *et al*. found GFP-WDR76 to be localized to nuclear foci [8]. Overall, the limited data from investigating both the yeast and human form of this protein suggests roles in chromatin biology and the DNA damage response.

In this study we focused on the human WDR76 protein and sought to determine its protein interaction network and analyze its recruitment to laser induced DNA damage. To begin, we affinity purified proteins from cell lines stably expressing HaloTag™-H2A, H2B, H4, and WDR76 and identified several proteins involved in DNA repair processes associated with each bait protein. An analysis of the proteins specifically associated with WDR76 suggested additional links to chromobox containing proteins like CBX1, CBX3, and CBX5. To assess both WDR76’s role in DNA-damage response and its association with heterochromatin enriched proteins, we induced DNA damage by UV irradiation and observed the subsequent changes in localization of key proteins by microscopy [11,12]. Here we report that WDR76 is rapidly and intensely recruited to regions of localized DNA damaged induced by UV-laser microirradiation. Moreover, we show that the CBX1 and CBX5 proteins form puncta in cells and co-localize with WDR76. Thus our study suggests that WDR76 protein plays a role in both chromatin biology and the DNA damage response.

## Materials and Methods

### Materials

Magne™ HaloTag^®^ magnetic affinity beads and HaloTag™TMRDirect ligand were purchased from Promega (Madison, WI). The following HaloTag™ clones from the Kazusa DNA Research Institute (Kisarazu, Chiba, Japan) were used: WDR76 (FHC25370), XRCC5 (FHC07775), RPA1 (FHC01462), CBX1 (FHC07438), CBX3 (FHC02188), CBX5 (FHC10519), and PARP1 (FHC01012). HEK293T cells (ATCC® CRL-11268™) were from ATCC (Manassas, VA) and Flp-In™-293 cells (AHO1112) were from Invitrogen™ (Carlsbad, CA). CLIP-tag™Cell505 ligand was from New England Biolabs (Ipswich, MA). Mouse anti-yH2A.X (phospho-Ser-139) antibody (DNA double strand break marker 05–636) was from EMD Millipore (Bellerica, MA). Alexa Fluor 488 Chicken Anti-Mouse IgG (A21200) was purchased from ThermoFisher Scientific (Waltham, MA).

#### Construction of vectors for protein expression in HEK293 cells

HaloTag™ clones were transferred from the original Flexi® vector, pFN21A, into the vector “Halo pcDNA5/FRT PacI PmeI” as described by Banks *et al*. [13]. Histone H2B, H2A and H4 ORFs were transferred from the original Flexi® vector pFC14K (C-terminal HaloTag™ expression vector), into the pcDNA5/FRT vector, using the restriction sites NheI/NotI. Selected ORFs coding for PARP1, XRCC5, RPA1, CBX1, CBX3, and CBX5 were cloned into a modified pCLIP-tag™-FLAG vector. pCLIP-FLAG was constructed by inserting a DNA fragment encoding a FLAG tag followed by the restriction site Sgf1 into the multiple cloning site of the pCLIP-tag™ vector from New England Biolabs (Ipswich, MA). We also constructed CLIP-WDR76 and Halo-RPA1 vectors for use in a reverse tag imaging experiment.

#### Cell culture and Stable Cell Lines

HEK293T cells were cultured in DMEM at 37°C in 5% CO2. All media for transient transfections were supplemented with 10% fetal bovine serum, and 2 mM Glutamax. HEK293FRT cells stably expressing Halo-WDR76, H2A-Halo, H2B-Halo and H4-Halo were constructed using the Flp-In™ system (ThermoFisher Scientific) according to the manufacturer’s instructions. All media for HEK293FRT stable cell lines were supplemented with 10% calf serum, 1x penicillin and 1x streptomycin.

#### Protein Purification

HEK293FRT cells stably expressing Halo-WDR76 or Histones H2A-Halo H2B-Halo and H4-Halo were grown to 90% confluency in 10–850 cm^2^ roller bottles. Nuclear extracts were prepared as described [14], with the exception of the use of 0.42 M NaCl instead of KCL, and the nuclear fraction was passed through a 26-gauge needle three times prior to centrifugation. Prior to Halo-tag purification, protein concentrations were determined for nuclear lysates to have consistent amounts of starting material. The nuclear lysate was incubated with beads prepared from 200 μl Magne™ HaloTag® bead slurry for 1 hr at 4°C. The beads were washed three times with buffer containing 25 mM Tris·HCl pH 7.4, 136 mM NaCl, 2.7 mM KCl and 0.05% Nonidet® P40. Bound proteins were eluted by incubating the beads with buffer containing 50 mM Tris·HCl pH 8.0, 0.5 mM EDTA and 0.005 mM DTT, 2 Units AcTEV™ Protease (ThermoFisher Scientific) for 1 hour at 25°C.

### Proteomic Analysis

Purified Halo-tagged proteins were TCA precipitated and digested with LysC and Trypsin as described previously [15]. RAW files were converted to the ms2 format using RAWDistiller v. 1.0, an in-house developed software. The ms2 files were subjected to database searching using SEQUEST (version 27 (rev.9) with no enzyme specificity considered [16] Tandem mass spectra were compared against 29,375 non-redundant human proteins obtained from the National Center for Biotechnology (2012-10-27 release). The database also included 176 common contaminant proteins, including human keratins, IgGs and peoteolytic enzymes. The database also included randomized versions of each nonredundant protein entry to estimate the false discovery rates. Spectra/peptide matches were filtered using DTASelect/CONTRAST [17] as described previously [15]. The number of spectra identified for each protein was used for calculating dNSAF values [18]. NSAF v7 (an in-house developed software) was used to create the final report on all non-redundant proteins detected across the different runs, estimate false discovery rates (FDR), and calculate their respective dNSAF values. All affinity purification data is available via ProteomeXchange with the identifier PXD003984 and MassIVE ID # MSV000079285 with the password josh1234 via the link (http://massive.ucsd.edu/ProteoSAFe/status.jsp?task=2ceca5bf2466476fbb3c652f46b90854).

#### Western Blotting Detection of WDR76 Associated Protein Candidates

HEK293FRT cells stably expressing Halo-WDR76 were generated as described in *Cell culture and Stable Cell Lines* section. Both the parental HEK293FRT and the stable cell line are cultured in regular Dulbecco's Modified Eagle Medium supplemented with 1x Gibco® GlutaMAX™ Supplement and 10% fetal bovine serum. Three confluent 150mm dishes of cells were collected and lysed with 900ul Mammalian Lysis Buffer (Promega) containing 1x Protease Inhibitor Cocktail (Promega), 1mM Dithiothreitol and 75U Benzonase Nuclease (Novagen). The cell lysate was homogenized by passing through 26-gauge needle 10 times and incubate at 4°C for 15min before centrifugation. The supernatant was brought up to 3ml with 1xTBS and incubated with 100ul of Magne™ HaloTag® bead slurry(Promega) overnight at 4°C. The beads were washed 5 times with 1xTBS containing 0.05% IGEPAL® CA-630. Proteins bound to the beads were eluted with 20U AcTEV Protease in 1x TEV Buffer (Invitrogen) for 1hr at 21°C. The collected protein samples were subjected to SDS-polyacrylamide gel electrophoresis and western blotting, 0.5% of the total cell lysate was loaded per well as Input and 10% of the total eluted protein was loaded per well as IP. Anti-Actin (ab3280) mouse monoclonal antibody, anti-XRCC5 (ab80592) rabbit monoclonal antibody and anti-WDR76 (ab108149) rabbit polyclonal antibody were purchased from Abcam. Anti-PARP1 (46D11) rabbit monoclonal antibody was purchased from Cell Signaling Technology. IRDye® 800CW Goat anti-Rabbit and IRDye® 680LT Goat anti-Mouse secondary antibodies were purchased from Li-cor.

To investigate possible interaction of WDR76 with components of heterochromatin 1 complex (CBX1, CBX3 and CBX5), we transiently transfected 293 cells stably expressing Halo WDR76 or HEK 293FRT (control) with 10 μg plasmid constructs encoding CLIP-FLAG tagged CBX1, CBX3 and CBX5 under a CMV promoter. Cells were incubated at 37°C, 5% for 48 hours and harvested. Halo purification was as described in HaloTag® Protein Purification System (Promega, G6280). Protease inhibitor (Promega, Catalog # G6521), Nonidet P40 Substitute Igepal CA-630 and DTT were added to the lysis buffer (Promega, Catalog # G9381). Following lysis, Benzonase® Nuclease (Millipore, Catalog # 70664–3) was added to each cell lysate. The lysates were incubated for 20minutes at room temperature with rotation. One milliliter of whole cell lysate obtained, 900μL was used for the purification and 100μL was kept aside as Input. Following TEV elution of WDR76, 1% of the whole cell lysate and 10% of the eluate was analyzed by SDS PAGE followed by Western blot on PVDF membrane. For detection, the membranes were incubated overnight in Mouse monoclonal anti-FLAG® M2 antibody (Sigma-Aldrich, Catalog Number A8592) and rabbit polyclonal anti Tubulin (ab15248). IRDye® 680LT Goat anti-Mouse IgG (LI-COR Biosciences, 926–68020) IRDye® 800CW Goat anti-rabbit IgG (LI-COR Biosciences,) were used for detection of CLIP FLAG tagged proteins and tubulin loading control).

### Topological Data Analysis

The topological network [19] first was built of 4 samples based on 164 varying proteins in the histone–WDR76 dataset using Z-scores and the Ayasdi platform (Menlo Park, CA). We used variance normalized Euclidean metric with resolution 30 and gain 3.0x. As a geometric filter function, we used Neighborhood Lenses 1 and 2. These lenses generate an embedding of high-dimensional data into two dimensions by embedding a k-nearest neighbor’s graph of the data. A k-nearest neighbors graph is generated by connecting each point to its nearest neighbors. All the nodes in the network are colored based on metric PCA coordinate 2. Unlike traditional network models where a single sample makes a single node, the size of a node in the topological network was proportional to the number of proteins with similar pattern.

### Microirradiation and Live Imaging

All live imaging and microirradiation experiments were performed on a PerkinElmer Life Sciences UltrVIEW VoX spinning disk microscope on a Carl Zeiss Axiovert 200M base. This microscope includes a yokagawa CSU-X11 spinning disk, an ORCA-R2 camera (Hamamatsu), and a PerkinElmer Life Sciences PhotoKinesis accessory. A 40x, 1.3 numerical aperture plan-apochromat objective was used. All experiments implemented a multiband dichroic (405/488/561/640 nm). The TMRDirect was excited with the 561-nm laser and imaged through a 415-476-nm, 580-650-nm multiband emission filter. The CLIP-Cell™ 505 was excited with the 488-nm laser and emission was collected through a 500-550-nm filter. Hoeschst was excited with the 405-nm laser and imaged through a 415-475-nm, 580-650-nm multiband emission filter. Laser power and exposure time were adjusted to maximize image quality and to limit photobleaching. All multicolor images and movies were collected in alternating excitation mode to eliminate cross-talk.

Prior to imaging or microirradiation, HEK293FRT cells stably expressing HaloTag™-WDR76 were plated at 40% confluency onto glass bottom MatTek culture dishes (35 mm, No. 2 14-mm diameter glass). Labeling proteins with the HaloTag™ TMRDirect ligand (Promega) involved adding the ligand to a final concentration of 100 nM, and allowing the cells to incubate overnight without media change. Co-localization experiments with CLIP-tag™ proteins were performed by transfecting HEK293FRT stably expressing Halo-WDR76 with the CLIP constructs as indicated above. HaloTag™ TMRDirect ligand was added directly to the cells to a final concentration of 100 nM and allowed to incubate overnight at 37°C in 5% CO2. The CLIP-Cell™ 505 ligand was added directly to the cells to a final concentration of 5 μM and allowed to incubate for 1 hr at 37°C in 5% CO2. The media was changed with OptiMEM to remove background fluorescence prior to imaging and microirradiation. Cells were stained with Hoeschst dye to mark nuclei and/or sensitize cells to UV irradiation 1 hr prior to imaging.

Microirradiation was performed as follows. Cells were subjected to laser UV microirradiation in a 1 μm x 9 μm stripe centered in the nucleus. The microirradiation was performed with 100% 405-nm laser power, and the stripe was exposed to 500–700 microwatts for ~5 s (100 iterations). Normal cell and nuclear morphology was preserved over the time scale of the experiment under these conditions. Quantitative analysis of protein recruitment following microirradiation was performed as described previously [12].

#### Microirradiation of Cells Treated with PARP1 Inhibitor

HEK293FRT cells stably expressing Halo-WDR76 were generated and cultured as described above. The cells were plated on 35mm glass bottom dishes (MatTek, No.1.5, collagen coated) and imaged at about 70% confluency. In order to visualize Halo-WDR76 proteins, HaloTag® R110DirectTM (Promega) were added to culture medium according to the manufacturer’s instruction. The PARP1 inhibitor KU-0058948 was dissolved in DMSO and added into culture medium at different concentrations 1hr before imaging. In order to observe Alc1 recruitments, cells were transfected with either mCherry or mCherry-Alc1 using FuGENE® 6 (Promega) 24 hours before PARP1 inhibitor treatments. mCherry and mCherry-Alc1plasmids are generously provided by Joan Conaway and Ronald Conaway’s lab. KU-0058948 was purchased from Axon MEDCHEM (Cat#2001, Batch No.2). The detailed microirradiation assay and image analysis was described in *Microirradiation and Live Imaging* section. As a sensitizer of 405-nm laser, the cells were stained with Hoechst 33258 (Sigma) prior to PARP1 inhibitor treatment. R110 was excited with the 488-nm laser and imaged with a 500-550-nm emission filter. mCherry was excited with the 561-nm laser and imaged through a 415-476-nm, 580-650-nm multiband emission filter.

#### Immunofluorescence

HEK293FRT cells stably expressing Halo-WDR76 were plated at 40% confluency onto gridded glass bottom MatTek culture dishes (35 mm, No. 2 14-mm diameter glass) and grown overnight. HaloTag™ TMRDirect ligand was added as described above. Prior to imaging Hoechst dye was added as described above. Cells were subjected to laser UV microirradiation as described, and the cells were fixed in 2% paraformaldehyde in ice-cold PBS for 20 minutes. After fixing, the cells were washed in ice-cold phosphate-buffered saline (PBS), permeabilized using 0.5% Triton in PBS, and incubated with blocking buffer containing 0.1% Triton X100, 100 μM MgCl_2_, 1.5% BSA in PBS for 1 hr. Cells were incubated with a mouse anti-yH2A.X (phospho-Ser-139) antibody in blocking buffer for 2 hr at room temperature (RT). Cells were then washed with blocking buffer 3 x 1 mL for 2 minutes each and incubated with an Alexa Fluor 488 Chicken Anti-Mouse IgG secondary antibody (1:200 dilution) for 2 hr at RT. Cells were washed with blocking buffer 2 x 1 mL for 2 minutes each, followed by PBS (2 x 1 mL for 3 minutes each). The PBS was aspirated off and the cells were allowed to sit at RT in the dark overnight. Cells were imaged the next day with the same imaging parameters described above.

## Results

### Topological Data Analysis of Histones and WDR76 Associated Proteins

Affinity purification followed by multidimensional protein identification technology (MudPIT) [20] was used to identify proteins associated either with the histones H2A, H2B, and H4, or with WDR76. These bait proteins were stably expressed in HEK293 Flp-in cells with an N-terminal HaloTag™ [21] on WDR76 and a C-terminal HaloTag™ on H2A, H2B and H4. Three biological replicates were analyzed for each of the bait proteins and distributed Normalized Spectral Abundance Factors (dNSAF) were determined to quantitate the relative amounts of the prey proteins identified with each bait [18] (S1 Table). Only the proteins that were present in at least 6 out of the 12 runs were retained (S2 Table). Next, an equal number of controls were performed to allow for the application of QSPEC [22] a method for determining the proteins specifically enriched in bait protein purifications over control purifications (S3 Table).

**Fig 1:**
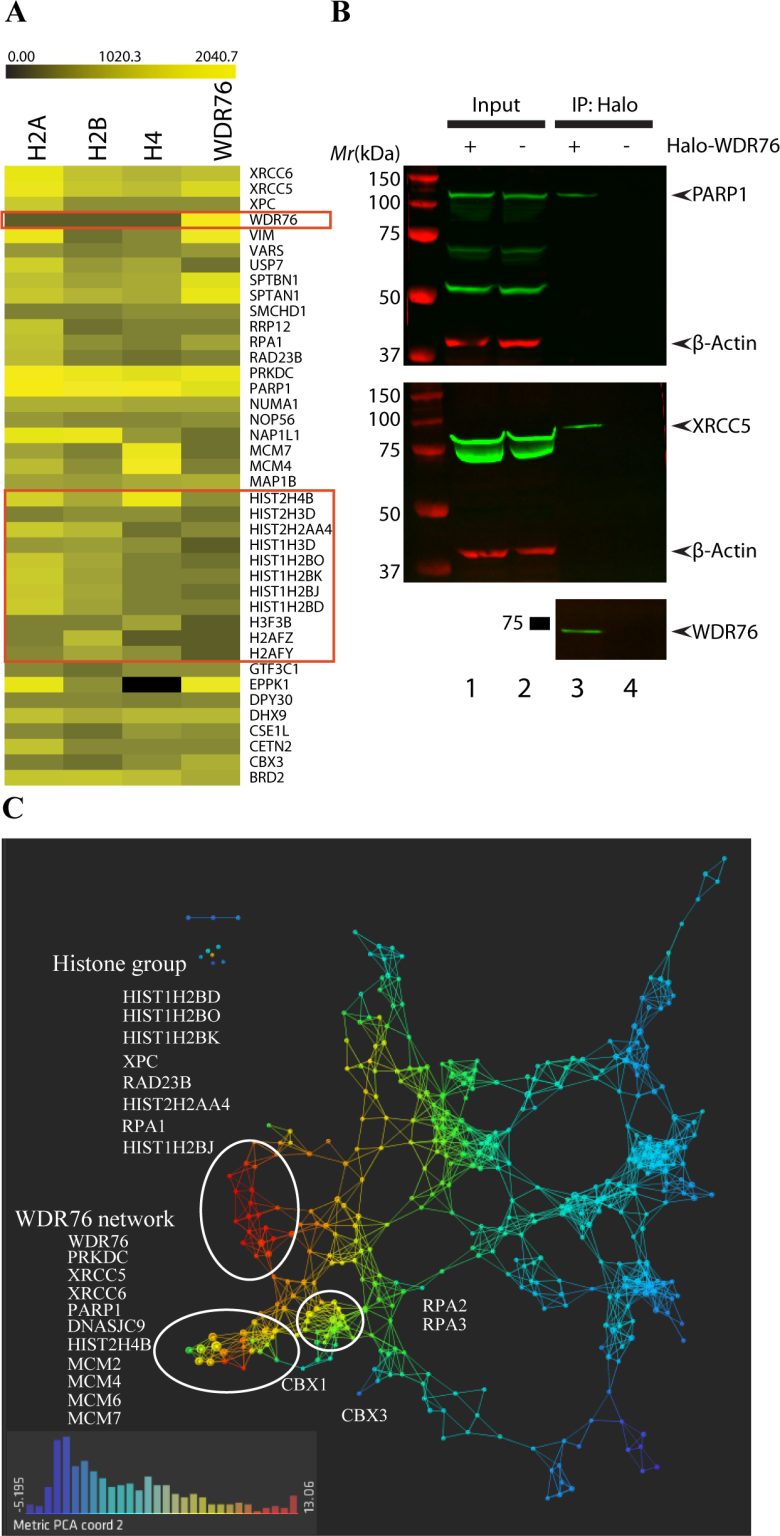
Quantitative Proteomic Analysis of Proteins Associated with WDR76 and Histones. (A) Heat map of relative abundance of 40 proteins shared between WDR76 and histones. The color intensity represents protein abundance with bright yellow displaying highest abundance (average distributed spectral counts) and black indicates that the protein was not detected in a particular purification. (B) Western blots of inputs and purifications from Halo-WDR76 stable cell line or HEK293FRT parental cell line shows that PARP1 and XRCC5 can be co-purified with WDR76, which is consistent with the proteomics analysis. Lane 1 and 2 are whole cell lysates from Halo-WDR76 stable cell line or HEK293FRT cells. Lane 3 and 4 are the protein purified with HaloTag™ magnetic beads from Halo-WDR76 stable cell line or HEK293FRT cells. The top panel was blotted with anti-PARP1 and anti-Actin antibodies; the middle panel was blotted with anti-XRCC5 and anti-Actin antibodies; the last panel was blotted with anti-WDR76 and anti- HaloTag™, which was not detected because of the cleavage during elution. (C) Topological data analysis was performed on 164 proteins with a Z-score greater than 2 in WDR76 and at least one histone purification compared to controls. All the nodes in the network are colored based on metric PCA coordinate 2. Filters with variance normalized Euclidean metric were used (resolution 30, gain 3.0x).

Previous analyses of GFP-WDR76 associated proteins reported interactions with OCR (SPIN1), HELLS, and GAN in one study [10], and HELLS, XRCC5, and XRCC6 in another study [8]. In agreement with these studies, we also found SPIN1, GAN, HELLS to be specifically enriched in Halo-WDR76 over histone associated proteins (S2 Table). In order to highlight the crosstalk between histones and WDR76 we represented the top 40 most abundant proteins in the data set in a heat map (Fig 1A and S3 Table). These proteins must be significantly enriched both in the WDR76 pulldown and in at least one histone purification (z-score> = 2) compared with control purifications (Fig 1A). Consistent with our previous results using the *S*. *cerevisiae* homolog of WDR76 [3], these results illustrate both that human core histones and WDR76 co-purify, and that they share proteins associations. Included in these associations were the DNA damage related proteins PARP1 and XRCC5, and western blot analysis of purified Halo-WDR76 confirmed these interactions (Fig 1B).

In addition, we constructed a topological map of WDR76-Histones associations in order to identify proteins in close proximity with WDR76 [19]. Topological Data Analysis (TDA) is a technique that enables us to easily and rapidly identify clusters of proteins [23]. In contrast to the traditional networks where a node corresponds to a protein, here a node consists of multiple proteins with similar patterns of protein association. The topological network reveals that WDR76 is in close proximity to DNA repair proteins like XRCC5, XRCC6, and PARP1 (Fig 1C). Other proteins detected in close proximity to WDR76 include the heterochromatin associated CBX1 and CBX3 proteins (Fig 1C).

### Recruitment of WDR76 to Sites of DNA Damage

**Fig 2:**
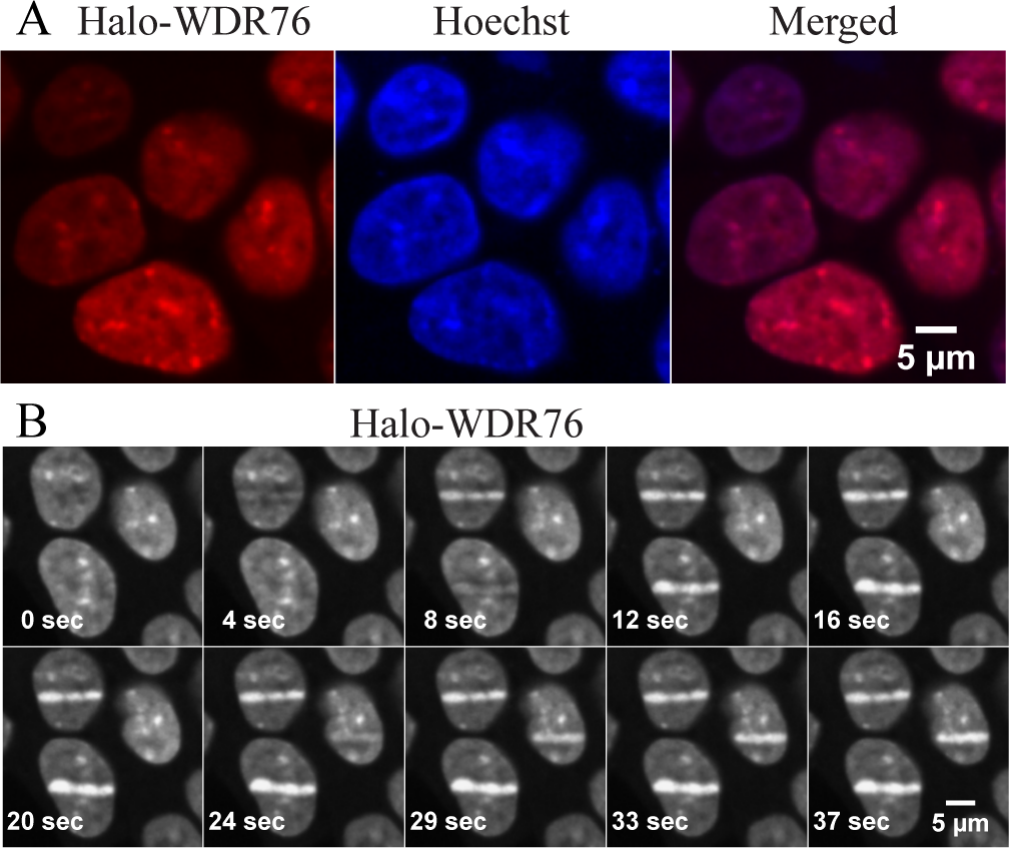
Localization of WDR76 Before and After DNA Damage. (A) HEK293FRT cells stably expressing Halo-WDR76 were analyzed showing nuclear localization. (B) UV microirradiation was induced in a stripe drawn across a live cell nucleus to follow the rapid recruitment of Halo-WDR76 to sites of DNA damage. Ten panels are shown ranging from 0 seconds (pre-laser) to 37 seconds after induction. During this time all three cells in the panel were damaged.

To systematically explore the potential role of WDR76 in DNA repair pathways, we constructed a biological pathway enrichment of the WDR76 associated proteins using the *DAVID* tool [24] (S1 Fig and S4 Table). For this analysis, we considered only proteins identified with high confidence that have both a z-score greater than 2 in the WDR76 pulldown over control and a FDR less than 0.05. Proteins involved in mismatch repair (*p* = 6.70E-08), nucleotide excision (*p* = 1.90E-07), DNA replication (*p* = 4.20E-04), and nonhomologous end joining (*p* = 7.20E-03) were highly enriched in the dataset (S1 Fig and S4 Table). These proteins, like RPA1, XRCC5, and PARP1, have roles in DNA repair, and so we asked whether we could detect the recruitment of WDR76 to sites of DNA damage in the nucleus (Fig 2). In order to initiate DNA damage, HEK29FRT cells, stably expressing Halo-WDR76, were subjected to UV laser microirradiation [11,12]. In these experiments, cells were treated with the TMRDirect™ Halo ligand for approximately 24 hours, and the nuclei treated with the DNA-interacting Hoechst dye for 1 hour, prior to imaging. Upon UV microirradiation with a 405-nm laser, we observed that WDR76 was intensely and rapidly recruited within seconds to sites of induced DNA damage (Fig 2B and S1 Movie).

**Fig 3:**
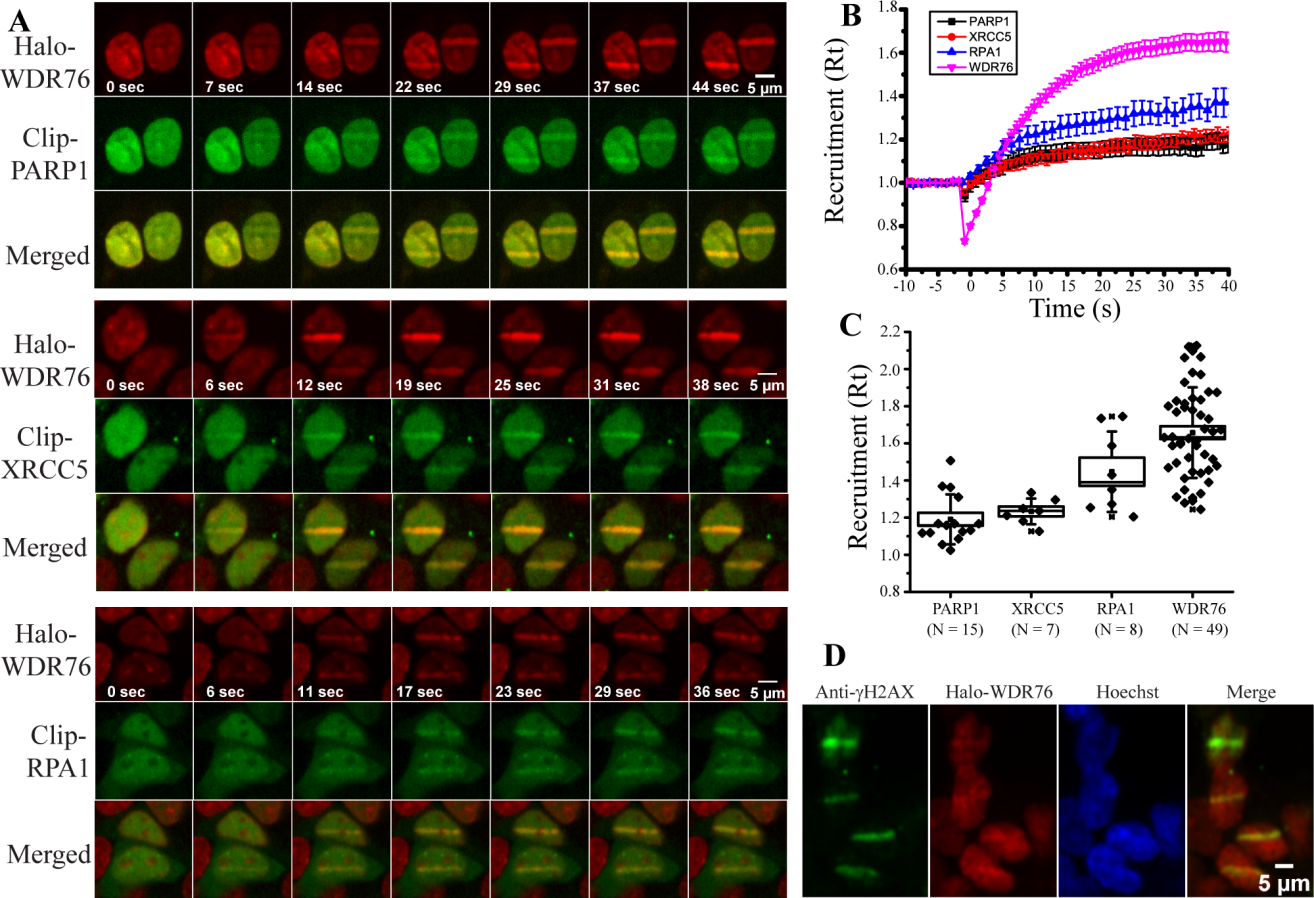
Co-localization of WDR76 with DNA Repair Proteins to Damaged Sites. (A) HEK293FRT cells stably expressing Halo-WDR76 and transiently expressing CLIP-PARP1, XRCC5 and RPA1 were stained with Hoechst dye, micro-irradiated with a 405 nm UV laser. Merged images of ligand labeled proteins after laser microirradiation are also provided. In cases where there is more than one cell in image the two cells were not damaged simultaneously. (B) Kinetics of recruitment to microirradiated regions of WDR76 (pink lines and symbols), XRCC5 (red), PARP1 (black) and RPA1 (blue). Cells were imaged every second, and intensity values were binned over 5-s intervals. Microirradiation was initiated at time = 0s. Averages are shown with the standard error. (C) Kinetics of recruitment to microirradiated regions of PARP1, XRCC5, RPA1 and WDR76 represented as box plots with the box showing the standard error with a coefficient of 1 and the whiskers showing the standard deviation with a coefficient of 1. (D) Enrichment of Halo-WDR76 at sites of localized DNA damage induced by UV laser microirradiation. HEK293FRT cells stably expressing Halo-WDR76 were stained with Hoechst dye, micro-irradiated with a 405 nm UV laser, fixed, and analyzed by indirect immunofluorescence using anti-γ-H2AX. The yellow line indicates site of co-enrichment.

We also assessed the co-recruitment of PARP1, XRCC5 and RPA1 with WDR76 to sites of DNA damage (Fig 3). We selected these proteins because they were significantly enriched in the pathway analysis of Halo-WDR76 pulldown (S1 Fig), and because they have well defined roles in DNA damage repair [25,26,27]. Constructs expressing PARP1, XRCC5 or RPA1 with an N-terminal Clip-tag™ [28] were co-transfected into HEK293FRT cells stably expressing Halo-WDR76. In addition to labeling Halo-WDR76 as before, we covalently labeled the Clip-tag™ proteins with a rhodamine derivative (Clip-Cell^TM^ 505), and added Hoechst dye to the cells an hour before imaging. All of these proteins were recruited to regions of the nucleus which had been irradiated to induce DNA damage (Fig 3A and S2–S4 Movies). When comparing the normalized recruitment (R(t)) of all four proteins over approximately forty seconds, WDR76 had the highest maximal R(t) followed by RPA1, XRCC5, and PARP1 (Fig 3C and 3D). This normalized recruitment of WDR76 is unlikely to be due to differences in expression level, since we observed no correlation between R(t) and expression level, as measured by relative fluorescence, for any of the proteins analyzed (S2A Fig). To determine if the recruitment of WDR76 was due to the tag being used, we carried out an experiment where Halo-RPA1 and CLIP-WDR76 were co-transfected into cells and their recruitment to laser induced damage analyzed (S3A Fig and S5 Movie). Both Halo-RPA1 and CLIP-WDR76 were recruited to damaged DNA and again WDR76 had a faster and higher maximal R(t) than RPA1 (S3B Fig).

Appearance of the phosphorylated histone H2AX (γ-H2AX) is known to be involved in the first steps of recruiting DNA repair proteins to the site of DNA damage and is a signature of double stranded break (DSB) repair [29]. Therefore we wanted to determine if γ-H2AX would co-localize with WDR76. We induced site-specific DNA damage by UV laser microirradiation as described above and observed co-localization of WDR76 with γH2AX (Fig 3D). Finally, to begin to try to dissect the mechanisms of recruitment to laser induced damage, we analyzed recruitment of WDR76 in cells treated with an inhibitor of PARP1, which had no discernable effect on recruitment (S4 Fig). In summary, our quantitative imaging studies demonstrate that WDR76 is rapidly and intensely recruited to laser induced sites of DNA damage.

### Co-Localization of WDR76 with CBX Proteins

**Fig 4:**
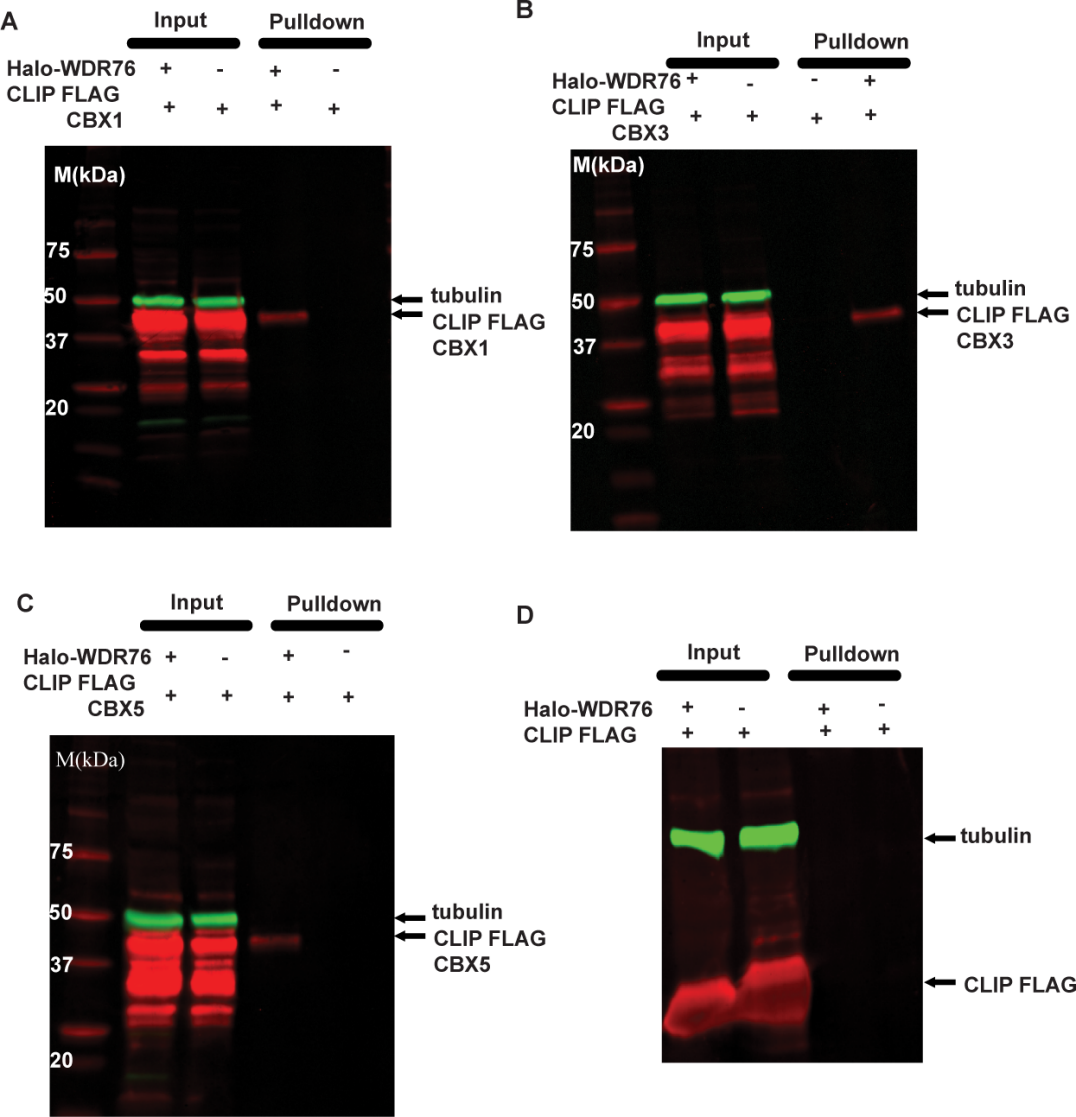
Co-immunoprecipitation of WDR76 with CBX1, CBX3, and CBX5. Cells stably expressing Halo-WDR76 were separately transiently transfected with CLIPtag™-FLAG proteins and HaloTag™ protein purifications conducted. Western blotting for tubulin was used as a loading control and western blotting with an anti-FLAG antibody was used to detect CBX1 (A), CBX3 (B), and CBX5 (C). Both the inputs and the results of the pulldown is shown. (D) Cells stably expressing Halo-WDR76 were transiently transfected with CLIPg™-FLAG construct to demonstrate that this construct was not responsible for the co-purifications of CBX proteins with WDR76.

**Fig 5:**
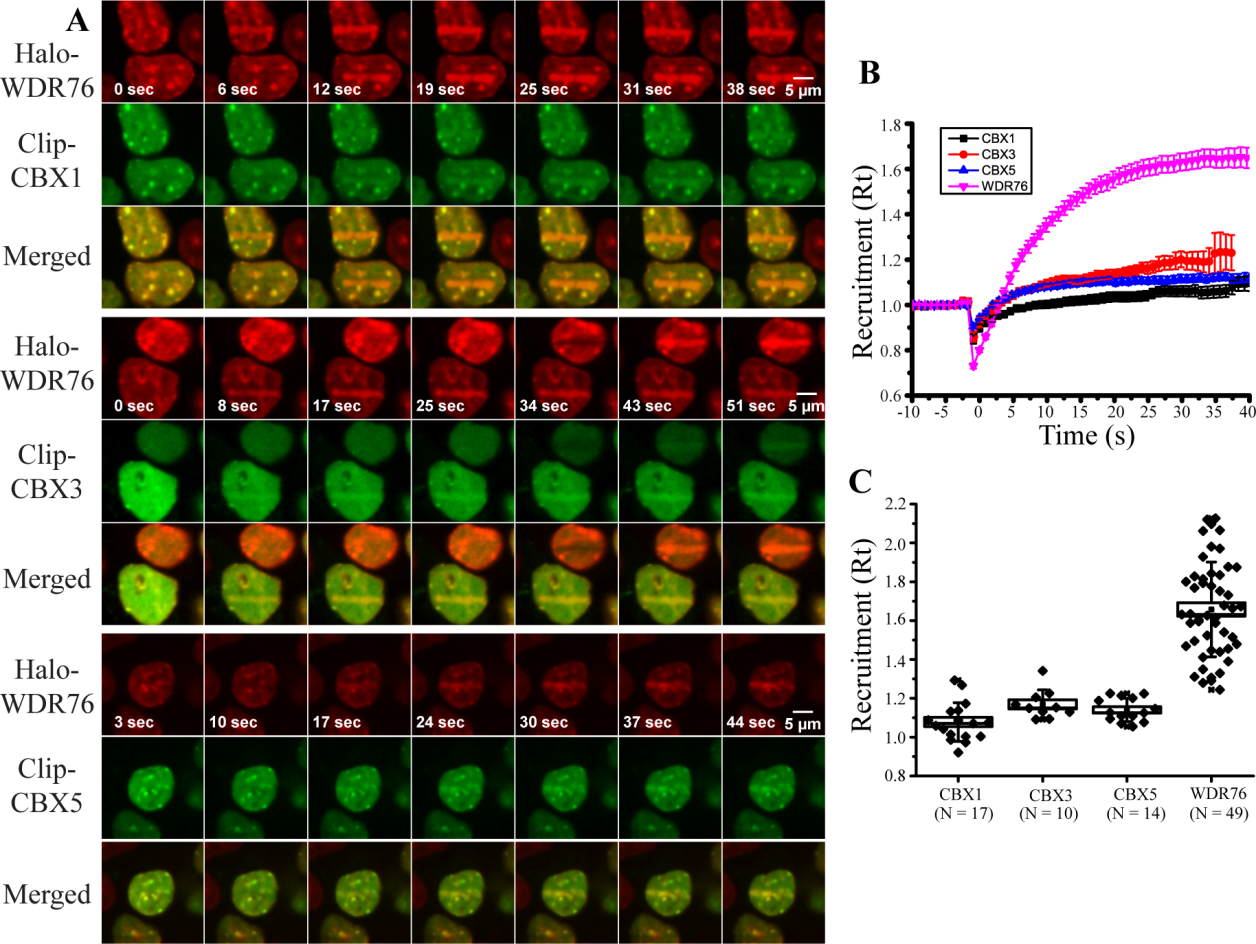
Co-localization of WDR76 with Heterochromatin Proteins. (A) HEK293FRT cells stably expressing Halo-WDR76 and transiently expressing CLIP-CBX1, CLIP-CBX3, and CLIP-CBX5 were stained with Hoechst dye, micro-irradiated with a 405 nm UV laser. Merged images of both labeled proteins after laser microirradiation are also shown. In cases where there is more than one cell in image the two cells not damaged simultaneously. (B) Kinetics of recruitment to microirradiated regions of WDR76 (pink), CBX1 (black), CBX3 (red), and CBX5 (blue). Cells were imaged every second, and intensity values were binned over 5-s intervals. Microirradiation was initiated at time = 0s. Averages are shown with the standard error. (C) Kinetics of recruitment to microirradiated regions of WDR76, CBX1 and CBX3 represented as box plots with the box showing the standard error with a coefficient of 1 and the whiskers showing the standard deviation with a coefficient of 1.

Next, we used the DAVID annotation tool [24] to determine the enrichment of protein domains in proteins associated with our Halo-WDR76 affinity purification (S5 Fig and S4 Table). The most highly enriched domain was the chromo shadow domain (*p* = 1.10E-04), which is an important domain in the HP1 proteins [30]. CBX1, CBX3, and CBX5 are the human homologues of HP1 proteins and have been shown to be recruited to sites of DNA damage [31]. CBX1 and CBX3 were also highlighted in the topological data analysis presented in Fig 1C. We first validated the affinity purification analysis of Halo-WDR76 with co-immunoprecipitation studies using CBX1, CBX3, and CBX5 (Fig 4). Cells stably expressing Halo-WDR76 were separately transiently transfected with CLIP-Flag-CBX1, CBX3, and CBX5 and all three proteins co-immunoprecipitated with WDR76 (Fig 4). Next, we used the same imaging approaches used to analyze the association of DNA damage proteins with WDR76 to analyze the association of WDR76 with CBX1, CBX3, and CBX5. Here, constructs expressing CBX1, CBX3 and CBX5 with an N-terminal CLIP-tag™ [28] were transiently co- transfected into cells stably expressing Halo-WDR76. Similarly to WDR76, we observed that CBX1, CBX3 and CBX5 were recruited to sites of UV-microirradiation induced DNA damage (Fig 5 and S6–S8 Movies). However WDR76 was recruited more rapidly and had a larger R(t) than any of the three CBX proteins (Fig 5). As was the case for RPA1, PARP1, and XRCC5, differences in relative fluorescence of WDR76, CBX1, CBX3, and CBX5 did not affect R(t) (S2B Fig). In addition, we observed that CBX1 and CBX5 form bright spots of fluorescence, or puncta (Fig 5A). These bright spots have been previously described as heterochromatic regions [32]. We identified the same puncta with Halo-WDR76, and they co-localize with the puncta resulting from expression of the CLIP-CBX1 and CBX3 proteins (Fig 4A). Together, these results support the association of WDR76 with CBX1, CBX3, and CBX5 and further demonstrate the rapid recruitment of WDR76 to sites of laser induced DNA damage.

## Discussion

Although DNA repair pathways have been identified and characterized, there are still proteins associated with DNA damage to be discovered. Previous studies in *S*. *cerevisiae* [3,4,6,7,8] suggested that CMR1/YDL156W plays roles in chromatin biology and the DNA damage response. The human homologue of this protein, WDR76 is even more poorly characterized, with studies suggesting it localizes to nuclear foci [8], associates with the Cul4-DDB1 ubiquitin ligase complex [9], and associates with 5-(hydroxyl)methylcytosine readers [10]. Affinity purified GFP-WDR76 was previously found to associate with OCR, HELLS, and GAN in one study [10], and with HELLS, XRCC5, and XRCC6 in a second study [8], but no additional validation of these associations was provided. The only information currently available on WDR76 beyond these interaction screens is that it forms some nuclear foci that may be enhanced upon treatment with the proteasome inhibitor MG132 or the damaging agent MMS, [8]. Based on our previous study of CMR1/YDL156w in *S*. *cerevisiae* [3], we sought to determine what proteins associated with WDR76 and histones and whether WDR76 is recruited to sites of DNA damage.

Using a combination of quantitative proteomics and quantitative imaging we found that WDR76 and the histones H2A, H2B, and H4 share many interactions that include proteins involved in the DNA damage response, including XRCC5, XRCC6, PARP1, RPA1, as well as proteins involved with chromatin biology, including CBX1,CBX3, and CBX6. The SPIN1, GAN, and HELLS proteins were highly enriched in WDR76 compared with histone affinity purifications, in agreement with prior GFP-WDR76 affinity purification data [8,10]. We validated the association of WDR76 with PARP1, XRCC5, CBX1, CBX3, and CBX5 using co-immunoprecipitation studies. Based on these results, we sought to determine whether WDR76 is recruited to sites of DNA damage in live cells.

To do this, we devised a method where HEK293 cells stably expressing Halo-WDR76 were analyzed using laser microirradiation induced DNA damage [11,12] with or without transiently transfected CLIP-tag™ proteins. We found that WDR76 was rapidly (within a second) and intensely recruited to sites of damage. We were also intrigued by the puncta seen in Halo-WDR76 expressing HEK293 cells; these puncta have been previously reported [8]. CBX1, CBX3, and CBX5 are the human homologues of the heterochromatin HP1 protein and have been shown to be recruited to sites of DNA damage [31]. Given the association of WDR76 with heterochromatin proteins we analyzed the pre- and post-DNA damage induced localization of CLIP-CBX1, CLIP-CBX3, and CLIP-CBX5 when separately transiently transfected into cells stably expressing Halo-WDR76. WDR76, CBX1, and CBX5 in particular co-localized to the puncta found in cells suggesting WDR76 may be co-localizing to sites of heterochromatin under normal conditions. In addition, while CBX1, CBX3, and CBX5 were also recruited to sites of DNA damage, WDR76 was again more rapidly and more intensely recruited than these proteins. Overall, by both co-localizing to heterochromatin and rapidly responding to DNA damage in cells, WDR76 may be an important protein in these two important biological systems, which warrants further investigation.

## Supporting Information

S1 Fig Biological pathways. (A) Pathway analysis was performed on proteins that passed a stringent criteria in WDR76 purification. The total number of pathways enriched was of 8 for KEGG pathways. Only results with a P-value <0.05 are shown. Proteins with a significant Z-score and FDR less than 0.05 were separated in DNA repair mechanisms. Proteins involved in mismatch repair are represented in (B). Proteins with a role in nucleotide excision repair are illustrated in (C). Proteins in NHEJ pathway are represented in (C). A FDR equal to zero was detected for proteins represented in (C).
(PDF)

S2 Fig Recruitment dependence on expression level. To determine if there is a strong expression dependence of r(t), per cell, the max value of r(t) is plotted vs relative fluorescence intensity (arbitrary units). While r(t) is comparable between different proteins, each protein is plotted on its own relative fluorescence scale. At the range of expression values examined, there is only a weak expression effect on r(t) for some of the proteins. The data presented here is from the same data points presented in Figs 3 and 5.
(PDF)

S3 Fig Tag Switching Experiment-Recruitment to DNA Damage of Halo-RPA1 and CLIP-WDR76. (A) HEK293T cells transfected with Halo-RPA1 and CLIP-WDR76. After 24hours, micro-irradiation of Hoechst treated cells was performed. Halo-RPA1 and CLIP-WDR76 are seen going to the laser damage site in the cell. (B) Graph showing the normalized fractional recruitment (R(t)) of two proteins, CLIP-WDR76 and Halo-RPA1 over approximately forty five seconds. CLIP-WDR76 was more strongly recruited than Halo-RPA1 to the damaged area in the cell.
(PDF)

S4 Fig The PARP1 inhibitor KU-0058948 does not affect WDR76 Recruitment. Neither intensity nor kinetics of WDR76 recruitment to microirradiation regions was affected by PARP1 inhibitor KU-0058948 concentrations up to 100uM; while the recruitment of Alc1, a chromatin remodeler of which recruitment and activation is dependent on PARP1 self-PARylation, was inhibited staring at 1uM. (A) The maximum recruitment of Halo-WDR76 to microirradiation regions at different PARP1 inhibitor concentrations. Values represent average ±SEM with n>10. (B) The maximum recruitments of mCherry or mCherry-Alc1 to microirradiation regions at different PARP1 inhibitor concentrations. Values represent average ±SEM with n>20. (C) Kinetics of Halo-WDR76 recruitment to microirradiation regions at different PARP1 inhibitor concentrations. Microirradiation was performed at time point 0 and images was taken at 1timepoint/sec. The curves were generated from an average of n>10 and the error bars stand for SEM.
(PDF)

S5 Fig Domain enrichment. Domain enrichment was performed on proteins that passed a stringent criteria in WDR76 purification. Proteins with the lowest p-values in Chromo domain are display in (B).
(PDF)

S1 Movie HaloWDR76 movie. A is a movie representative of the montage presented in Fig 2B.
(MOV)

S2 Movie CLIP-XRCC5_Halo-WDR76 movie. A movie representative of the montage presented in Fig 3A with Halo-WDR76 and CLIP-XRCC5.
(MOV)

S3 Movie CLIP-RPA1_Halo-WDR76 movie. A movie representative of the montage presented in Fig 3A with Halo-WDR76 and CLIP-RPA1.
(MOV)

S4 Movie CLIP-PARP1_Halo-WDR76 movie. A movie representative of the montage presented in Fig 3A with Halo-WDR76 and CLIP-PARP1.
(MOV)

S5 Movie Halo-RPA1_CLIP-WDR76. A movie representative of the montage presented in S3 Fig with Halo-RPA1 and CLIP-WDR76.
(MOV)

S6 Movie CLIP-CBX1_Halo-WDR76. A movie representative of the montage presented in Fig 5A with Halo-WDR76 and CLIP-CBX1.
(MOV)

S7 Movie CLIP-CBX3_Halo-WDR76. A movie representative of the montage presented in Fig 5A with Halo-WDR76 and CLIP-CBX3.
(MOV)

S8 Movie CLIP-CBX5_Halo-WDR76. A movie representative of the montage presented in Fig 5A with Halo-WDR76 and CLIP-CBX5.
(MOV)

S1 Table List of proteins detected in the histone and WDR76 stably expressing cell affinity purifications. (XLSX)

S2 Table List of proteins present in at least six of the twelve affinity purifications. (XLSX)

S3 Table Qspec results and list of top 40 proteins found in all histone and WDR76 stably expressing cell affinity purifications. (XLSX)

S4 Table Pathways analysis and domain analysis results. (XLSX)

## References

[1] Lindahl T, Barnes DE. Repair of endogenous DNA damage. Cold Spring Harbor symposia on quantitative biology. 2000;65:127–33. . 12760027 10.1101/sqb.2000.65.127

[2] Jackson SP, Bartek J. The DNA-damage response in human biology and disease. Nature. 2009;461(7267):1071–8. 10.1038/nature08467 . 19847258 PMC2906700

[3] Gilmore JM, Sardiu ME, Venkatesh S, Stutzman B, Peak A, Seidel CW, et al Characterization of a highly conserved histone related protein, Ydl156w, and its functional associations using quantitative proteomic analyses. Molecular & cellular proteomics: MCP. 2012;11(4):M111 011544. Epub 2011/12/27. 10.1074/mcp.M111.011544 M111.011544 [pii]. . 22199229 PMC3322567

[4] Choi DH, Kwon SH, Kim JH, Bae SH. Saccharomyces cerevisiae Cmr1 protein preferentially binds to UV-damaged DNA in vitro. Journal of microbiology. 2012;50(1):112–8. Epub 2012/03/01. 10.1007/s12275-012-1597-4 . 22367945

[5] Jones JW, Singh P, Govind CK. Recruitment of Saccharomyces cerevisiae Cmr1/Ydl156w to Coding Regions Promotes Transcription Genome Wide. PloS one. 2016;11(2):e0148897 Epub 2016/02/06. 10.1371/journal.pone.0148897 . 26848854 PMC4744024

[6] Tkach JM, Yimit A, Lee AY, Riffle M, Costanzo M, Jaschob D, et al Dissecting DNA damage response pathways by analysing protein localization and abundance changes during DNA replication stress. Nature cell biology. 2012;14(9):966–76. Epub 2012/07/31. 10.1038/ncb2549 . 22842922 PMC3434236

[7] Abu-Jamous B, Fa R, Roberts DJ, Nandi AK. Yeast gene CMR1/YDL156W is consistently co-expressed with genes participating in DNA-metabolic processes in a variety of stringent clustering experiments. Journal of the Royal Society, Interface / the Royal Society. 2013;10(81):20120990 Epub 2013/01/26. 10.1098/rsif.2012.0990 . 23349438 PMC3627109

[8] Gallina I, Colding C, Henriksen P, Beli P, Nakamura K, Offman J, et al Cmr1/WDR76 defines a nuclear genotoxic stress body linking genome integrity and protein quality control. Nature communications. 2015;6:6533 10.1038/ncomms7533 . 25817432 PMC4389229

[9] Higa LA, Wu M, Ye T, Kobayashi R, Sun H, Zhang H. CUL4-DDB1 ubiquitin ligase interacts with multiple WD40-repeat proteins and regulates histone methylation. Nature cell biology. 2006;8(11):1277–83. Epub 2006/10/17. 10.1038/ncb1490 . 17041588

[10] Spruijt CG, Gnerlich F, Smits AH, Pfaffeneder T, Jansen PW, Bauer C, et al Dynamic readers for 5-(hydroxy)methylcytosine and its oxidized derivatives. Cell. 2013;152(5):1146–59. Epub 2013/02/26. 10.1016/j.cell.2013.02.004 . 23434322

[11] Trautlein D, Deibler M, Leitenstorfer A, Ferrando-May E. Specific local induction of DNA strand breaks by infrared multi-photon absorption. Nucleic acids research. 2010;38(3):e14 10.1093/nar/gkp932 . 19906733 PMC2817483

[12] Weems JC, Slaughter BD, Unruh JR, Hall SM, McLaird MB, Gilmore JM, et al Assembly of the Elongin A Ubiquitin Ligase Is Regulated by Genotoxic and Other Stresses. The Journal of biological chemistry. 2015;290(24):15030–41. 10.1074/jbc.M114.632794 . 25878247 PMC4463447

[13] Banks CA, Boanca G, Lee ZT, Florens L, Washburn MP. Proteins interacting with cloning scars: a source of false positive protein-protein interactions. Scientific reports. 2015;5:8530 Epub 2015/02/24. 10.1038/srep08530 . 25704442 PMC4336944

[14] Dignam JD, Martin PL, Shastry BS, Roeder RG. Eukaryotic gene transcription with purified components. Methods Enzymol. 1983;101:582–98. Epub 1983/01/01. . 6888276 10.1016/0076-6879(83)01039-3

[15] Sardiu ME, Smith KT, Groppe BD, Gilmore JM, Saraf A, Egidy R, et al Suberoylanilide hydroxamic acid (SAHA)-induced dynamics of a human histone deacetylase protein interaction network. Molecular & cellular proteomics: MCP. 2014;13(11):3114–25. Epub 2014/07/31. 10.1074/mcp.M113.037127 . 25073741 PMC4223495

[16] Eng JK, McCormack AL, Yates JR 3rd. An approach to correlate tandem mass spectral data of peptides with amino acid sequences in a protein database. J Am Soc Mass Spectrom. 1994;5:976–89. 10.1016/1044-0305(94)80016-2 24226387

[17] Tabb DL, McDonald WH, Yates JR 3rd. DTASelect and Contrast: tools for assembling and comparing protein identifications from shotgun proteomics. J Proteome Res. 2002;1(1):21–6. . 12643522 10.1021/pr015504q PMC2811961

[18] Zhang Y, Wen Z, Washburn MP, Florens L. Refinements to label free proteome quantitation: how to deal with peptides shared by multiple proteins. Analytical chemistry. 2010;82(6):2272–81. Epub 2010/02/20. 10.1021/ac9023999 . 20166708

[19] Lum PY, Singh G, Lehman A, Ishkanov T, Vejdemo-Johansson M, Alagappan M, et al Extracting insights from the shape of complex data using topology. Scientific reports. 2013;3:1236 10.1038/srep01236 . 23393618 PMC3566620

[20] Washburn MP, Wolters D, Yates JR 3rd. Large-scale analysis of the yeast proteome by multidimensional protein identification technology. Nat Biotechnol. 2001;19(3):242–7. Epub 2001/03/07. 10.1038/85686 . 11231557

[21] Daniels DL, Mendez J, Mosley AL, Ramisetty SR, Murphy N, Benink H, et al Examining the complexity of human RNA polymerase complexes using HaloTag technology coupled to label free quantitative proteomics. J Proteome Res. 2012;11(2):564–75. Epub 2011/12/14. 10.1021/pr200459c . 22149079

[22] Choi H, Fermin D, Nesvizhskii AI. Significance analysis of spectral count data in label-free shotgun proteomics. Molecular & cellular proteomics: MCP. 2008;7(12):2373–85. Epub 2008/07/23. 10.1074/mcp.M800203-MCP200 . 18644780 PMC2596341

[23] Sardiu ME, Gilmore JM, Groppe BD, Herman D, Ramisetty SR, Cai Y, et al Conserved abundance and topological features in chromatin-remodeling protein interaction networks. EMBO reports. 2015;16(1):116–26. 10.15252/embr.201439403 . 25427557 PMC4304735

[24] Dennis G Jr., Sherman BT, Hosack DA, Yang J, Gao W, Lane HC, et al DAVID: Database for Annotation, Visualization, and Integrated Discovery. Genome biology. 2003;4(5):P3 Epub 2003/05/08. . 12734009 PMC193660

[25] Edwards MJ, Taylor AM. Unusual levels of (ADP-ribose)n and DNA synthesis in ataxia telangiectasia cells following gamma-ray irradiation. Nature. 1980;287(5784):745–7. Epub 1980/10/23. . 7432491 10.1038/287745a0

[26] He Z, Henricksen LA, Wold MS, Ingles CJ. RPA involvement in the damage-recognition and incision steps of nucleotide excision repair. Nature. 1995;374(6522):566–9. 10.1038/374566a0 . 7700386

[27] Taccioli GE, Gottlieb TM, Blunt T, Priestley A, Demengeot J, Mizuta R, et al Ku80: product of the XRCC5 gene and its role in DNA repair and V(D)J recombination. Science. 1994;265(5177):1442–5. . 8073286 10.1126/science.8073286

[28] Stagge F, Mitronova GY, Belov VN, Wurm CA, Jakobs S. SNAP-, CLIP- and Halo-tag labelling of budding yeast cells. PloS one. 2013;8(10):e78745 10.1371/journal.pone.0078745 . 24205303 PMC3808294

[29] Paull TT, Rogakou EP, Yamazaki V, Kirchgessner CU, Gellert M, Bonner WM. A critical role for histone H2AX in recruitment of repair factors to nuclear foci after DNA damage. Current biology: CB. 2000;10(15):886–95. . 10959836 10.1016/s0960-9822(00)00610-2

[30] Aasland R, Stewart AF. The chromo shadow domain, a second chromo domain in heterochromatin-binding protein 1, HP1. Nucleic acids research. 1995;23(16):3168–73. Epub 1995/08/25. . 7667093 10.1093/nar/23.16.3168 PMC307174

[31] Dinant C, Luijsterburg MS. The emerging role of HP1 in the DNA damage response. Molecular and cellular biology. 2009;29(24):6335–40. Epub 2009/10/07. 10.1128/MCB.01048-09 . 19805510 PMC2786877

[32] Nielsen AL, Oulad-Abdelghani M, Ortiz JA, Remboutsika E, Chambon P, Losson R. Heterochromatin formation in mammalian cells: interaction between histones and HP1 proteins. Molecular cell. 2001;7(4):729–39. . 11336697 10.1016/s1097-2765(01)00218-0

