# Supplementary figures and images for: WDR76 Co-Localizes with Heterochromatin Related Proteins and Rapidly Responds to DNA Damage
Source: PLoS One. 2016 Jun 1;11(6):e0155492. doi: 10.1371/journal.pone.0155492 (PMC4889050; doi:10.1371/journal.pone.0155492)

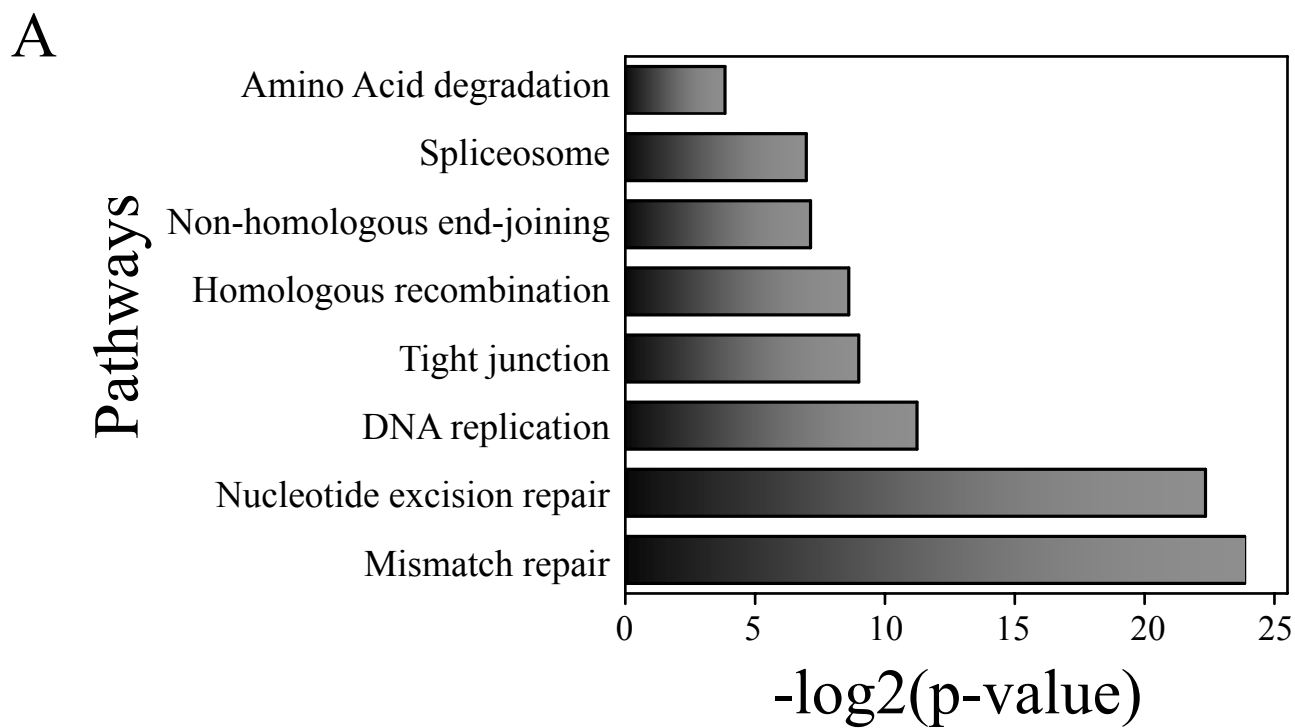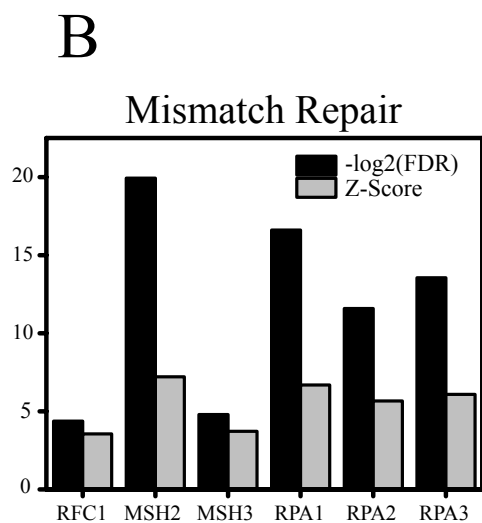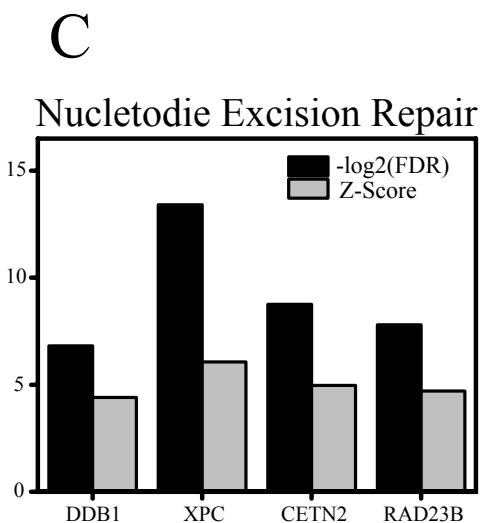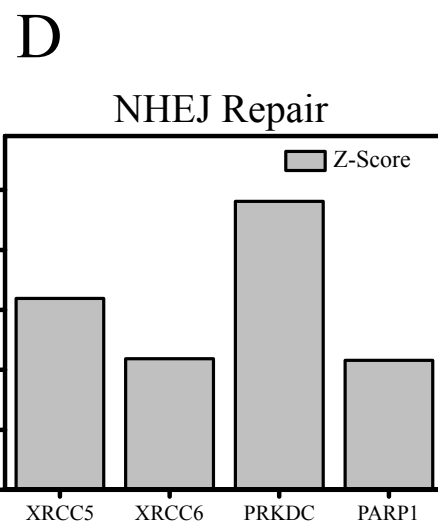

S1 Fig

Supplement: S1 Fig — (A) Pathway analysis was performed on proteins that passed a stringent criteria in WDR76 purification. The total number of pathways enriched was of 8 for KEGG pathways. Only results with a P-value <0.05 are shown. Proteins with a significant Z-score and FDR less than 0.05 were separated in DNA repair mechanisms. Proteins involved in mismatch repair are represented in (B). Proteins with a role in nucleotide excision repair are illustrated in (C). Proteins in NHEJ pathway are represented in (C). A FDR equal to zero was detected for proteins represented in (C). (PDF) [file pone.0155492.s001.pdf]

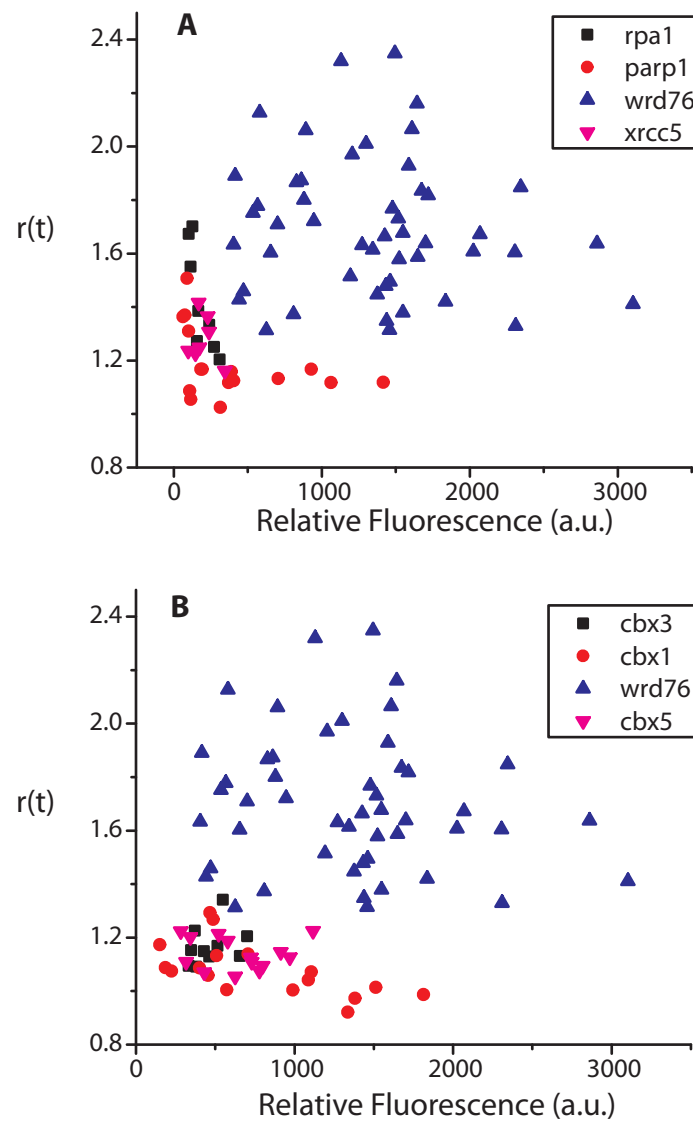

S2 Fig

Supplement: S2 Fig — To determine if there is a strong expression dependence of r(t), per cell, the max value of r(t) is plotted vs relative fluorescence intensity (arbitrary units). While r(t) is comparable between different proteins, each protein is plotted on its own relative fluorescence scale. At the range of expression values examined, there is only a weak expression effect on r(t) for some of the proteins. The data presented here is from the same data points presented in Figs 3 and 5. (PDF) [file pone.0155492.s002.pdf]

**A**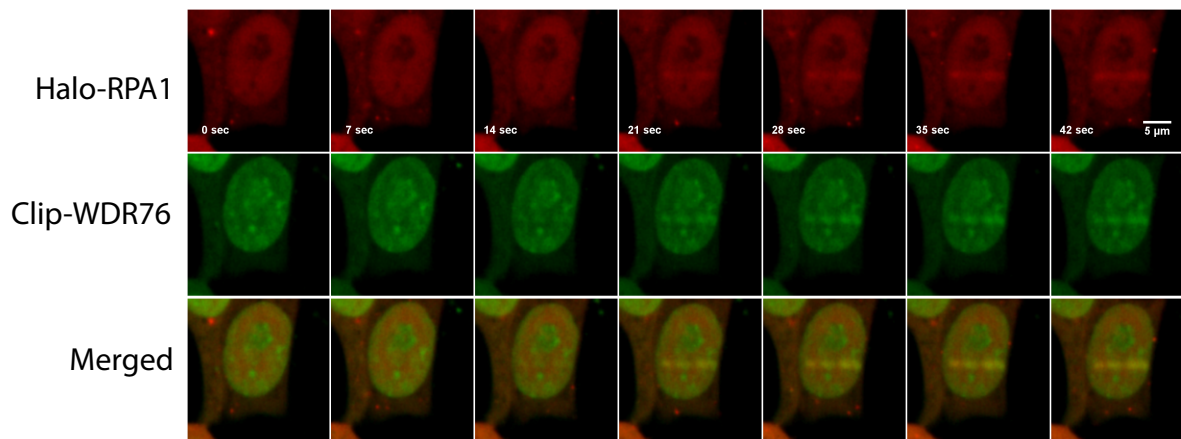**B**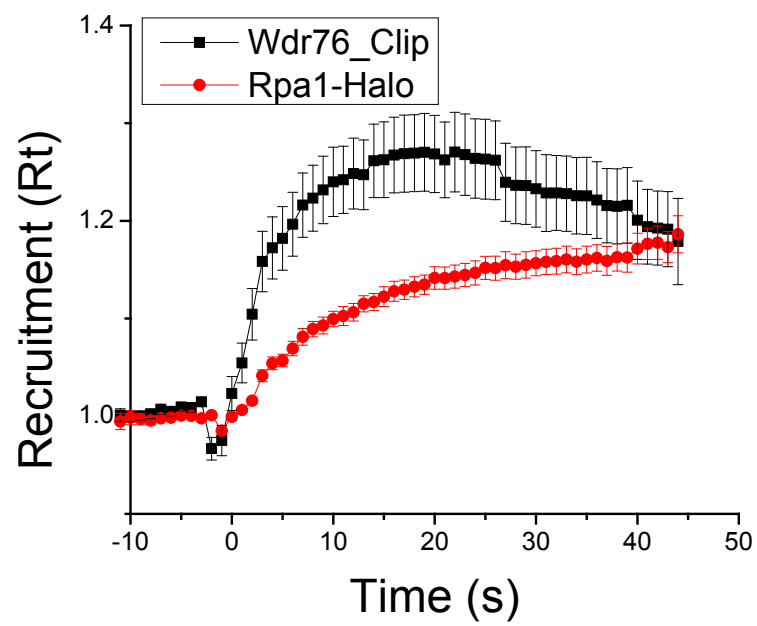

Supplement: S3 Fig — (A) HEK293T cells transfected with Halo-RPA1 and CLIP-WDR76. After 24hours, micro-irradiation of Hoechst treated cells was performed. Halo-RPA1 and CLIP-WDR76 are seen going to the laser damage site in the cell. (B) Graph showing the normalized fractional recruitment (R(t)) of two proteins, CLIP-WDR76 and Halo-RPA1 over approximately forty five seconds. CLIP-WDR76 was more strongly recruited than Halo-RPA1 to the damaged area in the cell. (PDF) [file pone.0155492.s003.pdf]

**A**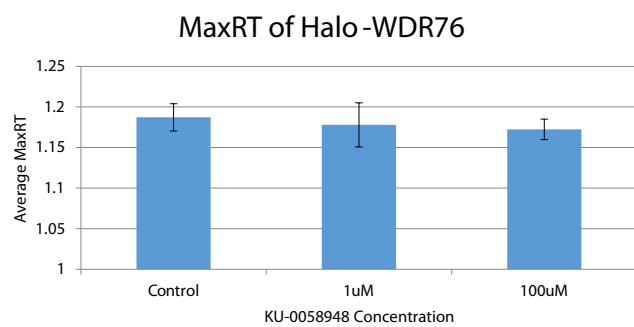**B**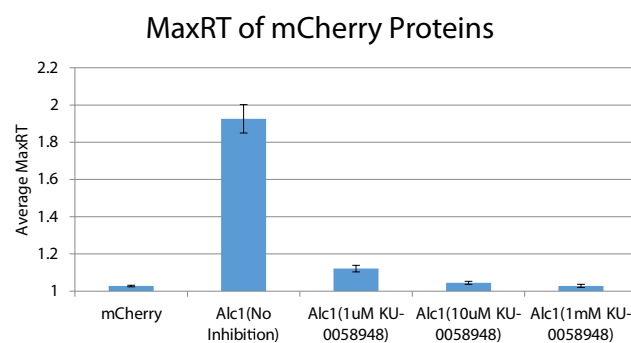**C**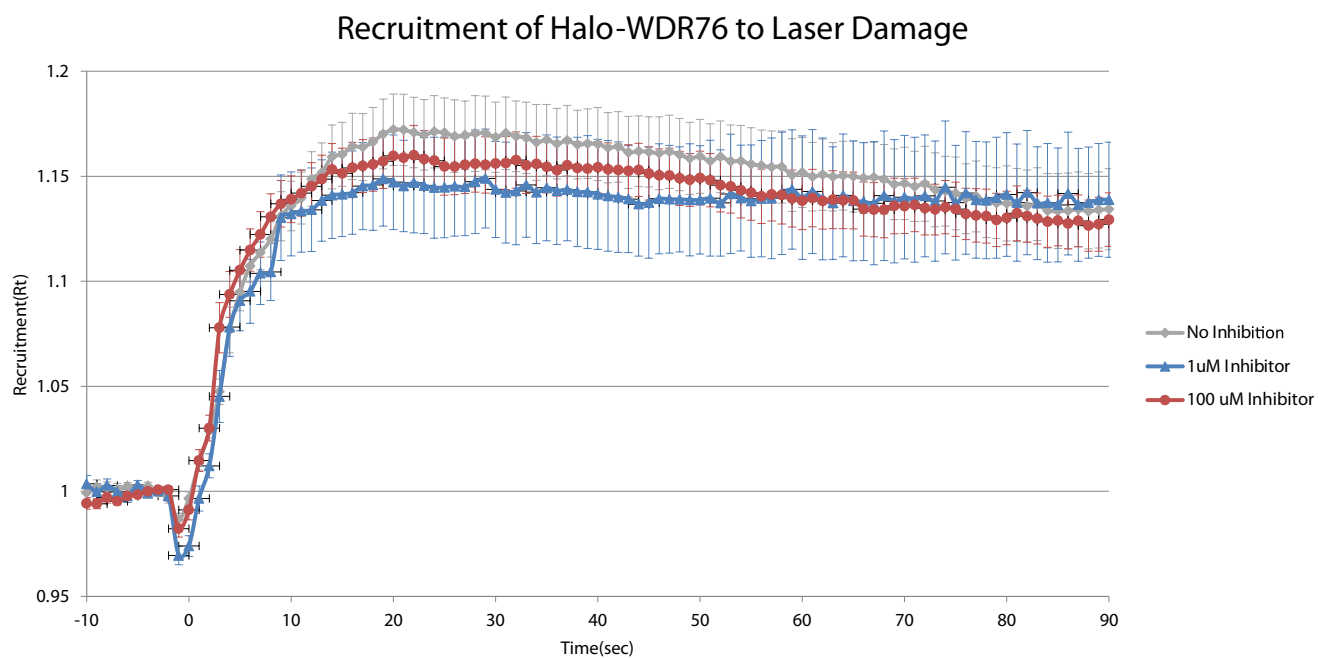

S4 Fig

Supplement: S4 Fig — Neither intensity nor kinetics of WDR76 recruitment to microirradiation regions was affected by PARP1 inhibitor KU-0058948 concentrations up to 100uM; while the recruitment of Alc1, a chromatin remodeler of which recruitment and activation is dependent on PARP1 self-PARylation, was inhibited staring at 1uM. (A) The maximum recruitment of Halo-WDR76 to microirradiation regions at different PARP1 inhibitor concentrations. Values represent average ±SEM with n>10. (B) The maximum recruitments of mCherry or mCherry-Alc1 to microirradiation regions at different PARP1 inhibitor concentrations. Values represent average ±SEM with n>20. (C) Kinetics of Halo-WDR76 recruitment to microirradiation regions at different PARP1 inhibitor concentrations. Microirradiation was performed at time point 0 and images was taken at 1timepoint/sec. The curves were generated from an average of n>10 and the error bars stand for SEM. (PDF) [file pone.0155492.s004.pdf]

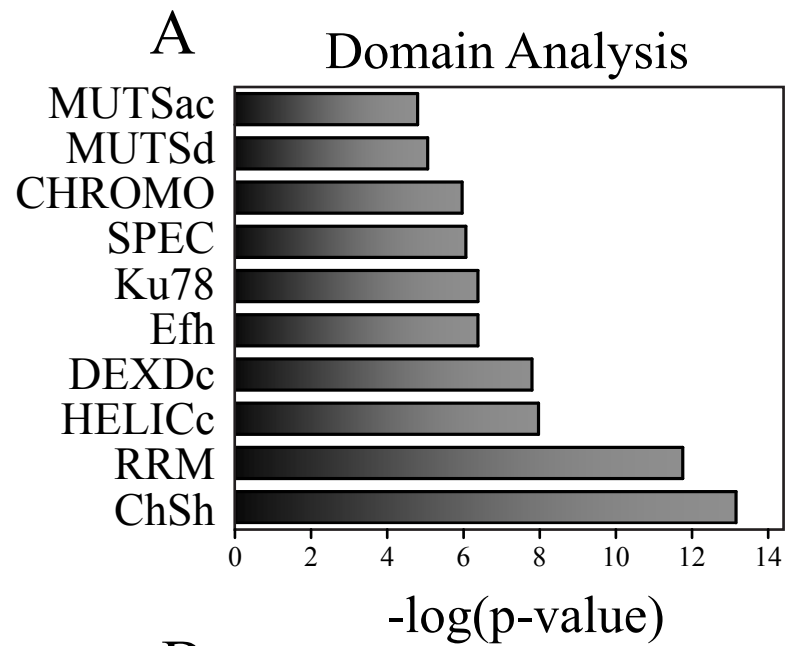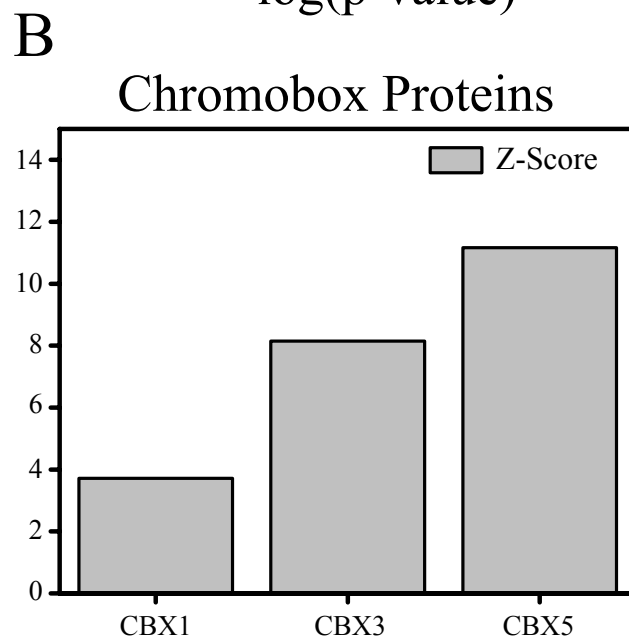

Supplement: S5 Fig — Domain enrichment was performed on proteins that passed a stringent criteria in WDR76 purification. Proteins with the lowest p-values in Chromo domain are display in (B). (PDF) [file pone.0155492.s005.pdf]
